# The effect of virtual and augmented reality training on soccer players: a systematic review of cognitive-motor performance

**DOI:** 10.5114/biolsport.2026.153531

**Published:** 2025-11-21

**Authors:** Ana Carolina Paludo, Adam Lipčák, Koulla Parpa, Elisavet Kyprianidou, Marios N. Avraamides

**Affiliations:** 1 Department of Psychology, University of Cyprus, Nicosia, Cyprus; 2 Department of Sport Performance and Exercise Testing, Faculty of Sports Studies, Masaryk University, Brno, Czech Republic; 3 Faculty of Sport and Exercise Science, UCLan University of Cyprus, Pyla, Cyprus; 4 CYENS Centre of Excellence, Nicosia, Cyprus

**Keywords:** Augmented reality, Soccer, Performance, Training, Virtual reality

## Abstract

This review provides a critical analysis of the findings from studies that investigated the effects of virtual reality (VR) and augmented reality (AR) training on the cognitive and sensorimotor skills of soccer players. A systematic search was conducted to identify studies reporting pre-post intervention outcomes related to (1) cognitive performance measured in traditional laboratory tasks, (2) cognitive-motor skills assessed with on-pitch tasks (e.g., heading), and (3) various self-report measures. The search was conducted in PubMed, Web of Science and SPORTDiscus databases, using the keywords *soccer* OR *football* AND *virtual reality* OR *augmented reality* AND *cognitive* OR *cognitive-motor* OR *self-report*, along with equivalent entry terms. Article selection followed the PRISMA 2020 guidelines. Of 289 records identified, 7 studies met the inclusion criteria. All included studies reported significant improvements in the VR and AR training groups, particularly in cognitive variables measured with reaction time and accuracy; cognitive-motor skills such as heading, passing, and shooting; and self-reported outcomes including perceived efficiency, self-confidence and sports engagement. One study also reported structural and functional brain adaptations following VR training. In conclusion, the findings of this systematic review support the potential of VR and AR training as an effective approach to improve cognitive-motor performance in soccer players. These results highlight that VR and AR can serve as effective tools in soccer training programmes, complementing traditional training. Open questions relating to the topic of the review are identified and are proposed as targets for future research.

## INTRODUCTION

Soccer is a dynamic and intermittent sport that requires a combination of physical, technical, tactical and cognitive skills to optimize performance during the competition [1–4]. While physical and technical-tactical aspects have been extensively investigated and systematically integrated into training periodization [5–7], cognitive training has received less emphasis in player development. However, as matches have become increasingly more complex and other skills have reached a high level of development, cognitive skills, particularly those involved in decision-making under time pressure, are now recognized as essential for optimal player performance and are considered key targets for development [4]. Despite this, cognitive training remains inconsistently incorporated into soccer periodization.

The complexity of a soccer match requires players to continuously engage in the process of analysing the situation, deciding to perform an action, and perceiving the outcome of the action, independent of the ball possession. To do so, a player must engage cognitive functions related to attention, perception, and working memory to effectively process vast amounts of information in a short time and make decisions [4, 8]. Thus, to be effective in improving player performance, cognitive training methodologies in soccer must align with the cognitive processes that underlie specific actions on the pitch. Thus, a clear understanding of which cognitive functions are involved in which tasks on the pitch is first required.

Although we are still far from having a complete understanding about the role of cognition in fast-paced sports such as soccer, trainers often use tasks during training aiming to improve reaction speed and decision-making in players, e.g., light training tasks, or reactive agility drills. Furthermore, recent technological advancements have allowed the development of a variety of digital tools that can be used to train cognitive skills. One example is the use of immersive training environments in virtual reality (VR) and augmented reality (AR) to simulate game-related context and provide players with opportunities to develop and refine cognitive functions under controlled yet realistic conditions. These technologies enable players to carry out tasks that engage cognitive processes such as visual scanning, orienting of attention, and responding with actions towards objects in controlled, yet realistic simulations [8, 9]. Consequently, VR and AR have become emerging tools in cognitive soccer training, offering a promising approach to improving players’ cognitive skills.

Although VR and AR-based training may offer a promising approach to improving players’ cognitive and sensorimotor skills, and despite the increasing adoption of VR and AR training programmes by professional clubs and leagues [10], several questions are yet to be addressed. For example, it is not yet clear how the various VR and AR tools engage different cognitive functions, whether they can produce transferable benefits, and whether realistic context is essential for this to happen. To provide a more nuanced understanding of these questions, the present systematic review aims to synthesize existing research on VR and AR training interventions in soccer, evaluating their effects on players’ cognitive and sensorimotor performance. The review will discuss the training characteristics that seem to be critical for achieving performance benefits, to provide a comprehensive understanding of how VR and AR protocols can be effectively integrated into soccer training periodization. Given the small number of published studies we found on the topic, our review follows the rapid review format.

## MATERIALS AND METHODS

The review was conducted under the Cochrane Rapid Reviews Guidance [11] and in alignment with the Preferred Reporting Items for Systematic Reviews and Meta-Analysis (PRISMA) guidelines [12].

### Eligibility criteria for selecting studies

The eligibility criteria were established based on the PICO criteria: Population (P): soccer players at all skill levels and from both genders; Intervention (I): participation in VR or AR training programmes. Comparator (C): studies assessing cognitive, perceptual and/or physical performance before and after a VR or AR training intervention; comparing an intervention group with alternative training or control groups. Outcomes (O): primary outcomes focused on changes in cognitive (e.g., decision-making, reaction time, attention.), cognitive-motor (e.g., heading, passing, shooting.) and/or self-report responses (e.g., enjoyment, effort, concentration.), while secondary outcomes included a detailed description of the training protocols (e.g., task type, session duration, frequency, intensity). Only original, short-communication, and case-study articles were included. Studies were excluded if they did not measure or report the outcomes of interest. Additionally, articles on American football, non-English publications, review papers, guidelines, conference abstracts and dissertations were not considered.

### Search strategy and selection process

A systematic search was conducted in MEDLINE (via PubMed), Web of Science and SPORTDiscus (via EBSCO) in February 2025. The search strategy was structured according to the PICO criteria and combined with the Boolean operators “AND” and “OR”. The following query was used: (“soccer” OR “football”) AND (“virtual reality” OR “virtual environment” OR “virtual system” OR “augment reality” OR “augment environment”) AND (“cognitive” OR “performance” OR “agility” OR “speed” OR “reaction” OR “reaction-time” OR “speedtime” OR “decision-making”).

All retrieved articles were imported into the Rayyan systematic review software [13] for screening and selection. One author was responsible for uploading the articles, removing duplicates and excluding non-English studies as well as those that did not meet the predefined illegibility criteria. Subsequently, two independent authors screened the titles and abstracts, and in case of disagreement, a third author was consulted. Full-text screening was then conducted by two authors, with one additional author verifying all excluded studies. Any discrepancies during this phase were resolved through discussion with the third author.

After finalizing the selection of the studies to include in the review, additional sources were identified by screening the reference lists of the included articles and the grey literature (e.g., Google Scholar).

### Data collection process

Data were extracted by two independent researchers. To facilitate systematic data extraction, the researchers developed a structured form to retain key information, including study characteristics, details of the VR or AR training interventions and the outcome measures before and after the intervention. In instances where information was incomplete, the corresponding authors of the articles were contacted. Subsequently, data were presented descriptively, displaying significant increases (↑), decreases (↓) or non-changes (↔) following the intervention. Furthermore, the findings were reported in terms of differences (↑, ↓, ↔) between groups that underwent VR or AR training compared to a control group or alternative training.

## RESULTS

### Included studies and characteristics

**FIG. 1:**
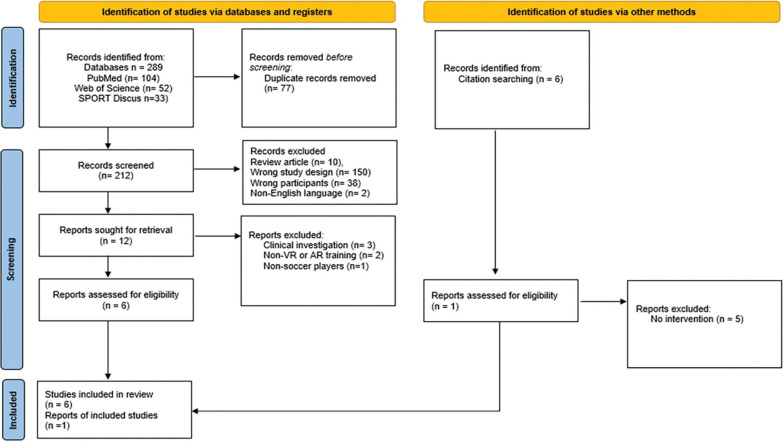
Flow chart diagram of the study selection process (PRISMA 2020)

A total of 289 records were identified across the searched databases. After removing duplicates, 212 records remained for screening. Of these, 200 studies were excluded due to methodological design, focus on other sports, or non-English language. In the final selection phase, 12 full-text articles were assessed. Six articles were retained for the review after excluding those focused on non-VR/AR training and clinical applications. An additional search on alternative sources carried out in March-April identified six potentially eligible studies. After full-text screening, only one met the inclusion criteria. Consequently, a total of seven studies were included in the final review (Figure 1).

Tables 1 and 2 present the details of the included studies. The studies were published between 2021 to 2024 and were conducted in Switzerland [14], Brazil [15], Turkey [16], the United Kingdom [17], Indonesia [18], the United States of America [19] and France [20]. Among these, two studies focused on elite [14] and national-level [15] soccer players, while the remaining four examined recreational [17], beginner [18], young football licensed [16], high school players [19] and a former amateur soccer player [20]. One study exclusively investigated female soccer players [19], while another [17] included both male and female players, albeit with a substantial imbalance in the design, i.e., 30 male vs. 6 female players.

**TABLE 1:** Characteristics and primary outcomes of included studies on professional soccer players.

Characteristics					Cognitive		Cognitive-Motor Skills	
Author, year (Country)	Sample (N), gender (M, F), age (yrs)	Level	VR or AR	Main Aim	Inhibitory control (accuracy and response time)	Visual Search (fixation: number and duration)	Kick (Redirection Threshold)	Passing Decision-Making
Bloechle et al., 2024 (Switzerland) [14]	N = 13, M 15.77 ± 0.73 yrs (U16 to U18)	Elite	AR	Penalty kick	–	–	↓	–

Fortes et al. 2021 (Brazil) [15]^*^	N = 26 M, 15.4 ± 0.3 yrs VR = 13 VID = 13	National level	VR	Decision-Making, Visual Search, Inhibitory Control	VR = ↑ VID = ↑ VR *vs* VID = ↔	VR = ↑ VID = ↑ VR *vs* VID = ↑	–	VR = ↑ VID = ↑ VR *vs* VID = ↑

**Note:** N = sample size; M = male; VR = virtual reality group; AR = augmented reality; CG = control group; MB = match broadcast; VID = video screen, RT = reaction time, SSG = small-sided game.

* Immersive reality.

**TABLE 2:** Characteristics and primary outcomes included studies on non-professional soccer players.

Characteristics					Cognitive		Self-Evaluation		Cognitive – Motor Skills			Neuroanatomical

Author, year (Country)	Sample (N), gender (M, F), age (yrs)	Level	VR or AR	Main Aim	RCS-PL	PLV	Efficacy and Confidence	SES	Heading accuracy	Heading Rate	Shooting and passing	Structural and Functional
Gürbüz and Taş 2023. (Turkey) [16]	N = 24, M 12–13 yrs, VR = 8 VRT = 8 CG = 8	Licensed Football Players	VR	Heading Training	–	–	–	–	–	VR = ↑ VRT = ↑ CG = ↔ VRT vs VR,CG = ↑^*^	–	–

Marshall et al. 2023.(UK) [17]	VR = 14 M, 4 F 24.17 ± 5.0 yrs CG = 16 M, 2 F 28.67 ± 5 yrs	Recreational	VR	Heading Training	–	–	VR = ↑ CG = ↔ VR *vs* CG = ↔	–	VR = ↔ CG = ↔ VR *vs* CG = ↔	VR = ↑ CG = ↔ VR *vs* CG = ↔	–	–

Rusmanto et al. 2023 (Indonesia) [18]	N = 40, M 16–20 yrs VR = 20 CG = 20	Beginner	VR	SES, Technical Skills	–	–	–	VR = ↑ CG = ↔ VR *vs* CG = ↑	–	–	VR = ↑ CG = ↔ VR vs CG = ↑	–

Wilkerson et al. 2024 (USA) [19]	N = 42, F 15.2 ± 1.2 yrs VR = 19 CG = 23	High School	VR	Injury Risk by Speed and Perception	VR = ↑ CG = ↑ VR *vs* CG = ↑	VR = ↑ CG = ↑ VR *vs* CG = ↔	–	–	–	–	–	–

Richlan 2025 France [20]	N = 1, M	Former amateur player	VR	Heading Training			–	–	–	↑	–	↑

**Note:** N = sample size; M = male; VR = virtual reality group; AR = augmented reality; RCS-PL = Rate Correct per Second – Perceptual Latency; PLV = Perceptual Latency Variability; SES = Sport Engagement; UK = United Kingdom; USA = United State America; CG = control group; VR = Virtual Reality group; VRT = Virtual Reality and Traditional training.

* Comparison of delta percentage of the change.

The primary outcomes evaluated across the included studies consisted of measures of cognitive performance (e.g., inhibitory control, visual search behaviour) obtained either through tasks that entailed sensorimotor actions (e.g., shooting, passing and heading) or computer-based cognitive tasks (e.g., the Stroop task). Some have also collected self-reported measures, e.g., confidence, self-efficacy, and reported sport engagement. Also, except for the study of Bloechle et al. [14] that used AR, all studies employed VR.

To preview our findings, pre- and post-intervention measurements demonstrated significant improvements following VR and AR training programmes, with most measures indicating greater benefits in the intervention than in the control or the alternative training groups.

### Interventions and outcomes

Most of the studies reviewed evaluated interventions that relied on graphics and entailed some form of user interaction, e.g., performing a sensorimotor task in VR. Also, most of them evaluated the effectiveness of the interventions by collecting pre- and post-intervention data from cognitive-motor tasks. A subset of studies analysed self-reported measurements, such as self-efficacy and confidence ratings.

The only study that used an intervention that did not entail any user interaction was conducted by Fortes et al. [15]. In this study, 26 young (15–16 years old) soccer players were assigned to two groups that underwent an 8-week training intervention that included watching videos either in a phone-based VR headset or on a desktop screen. For both groups, the intervention included watching 60 video clips weekly, depicting from a first-person perspective 4 individuals as teammates and 5 individuals as opponents performing variations of two different offensive patterns. The players’ passing decision-making and visual search behaviour were assessed during 5 vs. 5 and 3 vs. 3 soccer matches, before and after the intervention. Passing decision-making was quantified based on a set of predetermined criteria (e.g., a passing decision was deemed appropriate if the ball was passed to an unmarked teammate). Visual search behaviour was deduced by recording the number and duration of gaze fixations, computed from eye-tracking data. In addition, inhibitory control was assessed with the Stroop task. Results from the study showed that both groups improved in terms of passing decision-making, number of fixations, and duration of fixation from pretest to post-test. However, the improvements were in all three measures larger for the VR group. Notably, in terms of inhibitory control, both groups improved from pre-test to post-test, but the improvement did not differ across groups.

All other studies used intervention tasks in VR that required some user interaction. For example, the study by Wilkerson et al. [19] employed an immersive VR test that required reaching and lunging towards a visual stimulus that moved horizontally on the display and computed a composite measure of accuracy and reaction time, i.e., the number of correct responses per second of response time. The study used a regression-discontinuity design, based on which participants – 50 female high-school soccer players – who performed lower than the median score were assigned to the training condition and those who performed higher than the median score were assigned to the control group. Over a period of 7 weeks, participants in the training group carried out short training sessions with the same task that was modified to induce user engagement. Then all participants underwent a post-training assessment that was identical to the one used to obtain baseline performance. The results showed that the training group improved in the VR task, reaching the level of the control group that started with higher scores. Notably, this improvement was observed in what the authors refer to as perceptual latency, a measure that considers only the period up to the initiation of the response, factoring out the execution of a sensorimotor response to the target. Thus, the findings of the study document that the improvement with the VR training in the group with sub-optimal baseline performance was due to improvements in perceptual and decision-making processes, rather than in sensorimotor processes.

One study used an intervention that involved AR rather than VR [14]. In this study, 13 young elite players from U16 and U17 teams in Switzerland trained in a session that required penalty kicks to be taken on an actual pitch against a holographic goalkeeper diving to save them. Virtual cues instructed the player where to direct the ball, creating conditions in which the kick had to be redirected, i.e., when the goalkeeper began to dive towards the cued side, the player had to suppress the initial plan and kick the ball to the other side. It was found that after 10 training sessions with 20 penalty kicks, the 50% redirection threshold (i.e., the minimum time required to successfully redirect the kick 50% of the time) was reduced by 120 ms. Thus, the study of Bloechle et al. [14] showed that training with an AR simulator improved the players’ ability to make quick redirection decisions and score from the penalty spot. That said, this study investigated whether players improved on the actual task they were trained with, as opposed to examining transfer to a separate dependent measure. In addition, this study involved no control condition.

Like the study of Bloechle et al. [14] that evaluated on-pitch performance, a study by Gürbüz & Taş [16] assessed the effectiveness of a VR intervention in improving heading skills. Players, aged 12 to 13, licensed by the Turkish Football Federation, were assigned to one of three groups: a VR-only group (n = 8), a control group that did not perform heading training (n = 8), and a combined VR and traditional heading training group (n = 8). Heading accuracy was assessed at three time points – pre-intervention, mid-intervention (week 4) and post-intervention – using an on-field test involving a ball throwing machine and target zones to measure players’ scoring precision. All participants engaged in a regular soccer training programme twice a week for eight weeks, including a soccer-specific warm-up, ball exercises, joint mobility and stretching, and cool-up routines. The VR intervention consisted of heading virtual balls projected by a simulated ball-throwing machine within an indoor soccer environment, progressing through five levels of difficulty. The results showed that both groups incorporating VR training demonstrated significant improvement in heading accuracy from pre- to post-intervention, with no significant difference at the mid-point. The control group showed no improvement across the intervention. Notably, the combined VR and traditional heading group achieved greater improvement than the VR-only group, suggesting that VR can enhance, but not replace, traditional on-field heading practice.

In addition to Bloechle et al. [14], a study by Marshall et al. [17] also involved heading training. In this study, 18 recreational-level players trained their heading in VR over a period of 7–10 days and were compared to 18 control participants who did not. The pre-test and post-test involved heading balls shot from a ball launcher on an indoor football pitch. The number of goals scored was recorded. Furthermore, video analysis software was used to quantify the accuracy of the shots by awarding points depending on how close the ball landed near the goal post, i.e., more points were awarded to shots near the post that were unlikely to be saved by a goalkeeper than those in the centre of the goal. The results showed that while the VR group scored more goals after the training intervention than before, the control group did not. No significant effects were found for accuracy. Notably, this study also included self-report measures of self-efficacy (i.e., a rating by players of the general heading ability) and self-confidence in scoring the headings. An increase in scores for both measures was observed in the VR group from the pre-test to the post-test. No increase was observed in the control group.

Self-reported measures were also examined in the study of Rusmanto et al. [18], which investigated the effect of a VR training intervention on sports engagement and soccer-related skills. Forty male beginner-level soccer players from Indonesia, aged 16 to 20 years, were assigned to either a VR training group (n = 20) or a traditional training control group (n = 20). Participants from both groups participated in football training three times per week over a 12-week period. The VR group engaged in simulated shooting and passing drills, while the control group only followed traditional football training. Sport engagement was assessed using a validated self-report scale that measured the sub-dimensions of vigour, dedication and absorption. Additionally, real-world passing ability and shooting to targets were evaluated. The results indicated that the VR training showed significantly higher levels of sport engagement compared to the control group and had a positive effect in improving both the passing and the shooting performance.

Finally, a recent study by Richlan [20] was the first to examine the effects of VR training on brain structure and function, as well as the impact of detraining. A 37-year-old former amateur soccer player, who had been inactive for 15 years, completed a 4-week VR heading training programme involving simulations of control, shooting, clearing and passing. Training was conducted six days per week for 30 minutes per session. During the intervention period, the player underwent weekly functional MRI (fMRI) scans to assess neural adaptations, while real-world heading performance – including ball control, heading accuracy and goal-scoring ability – was evaluated at pre- and post-intervention time points. The results showed improvements in heading performance and neuroplasticity, including increased grey matter volume in the left thalamus, white matter volume in the cerebellum, and cortical thickness in regions related to motor control, visual processing and memory. Functional connectivity also increased across several brain networks. Importantly, these improvements were retained four weeks after training, demonstrating a lasting effect of the VR intervention.

## DISCUSSION

This systematic review aimed to summarize the effects of virtual (VR) and augmented reality (AR) training on the cognitive-motor performance of soccer players. Across the included studies, the main finding is that VR and AR interventions significantly improve cognitive and cognitive-motor performance, highlighting the positive impact of using these technologies. Notably, improvements were observed even with interventions that included as few as three sessions and with short sessions lasting five minutes.

A common finding across the studies we reviewed was that greater improvements were observed in tasks that closely resembled the training interventions than in tasks that did not. For example, studies that implemented heading training in virtual environments also demonstrated improvements in real-world heading performance. Players not only improved their scores on heading tests in the pitch [16, 17, 20], but also enhanced the accuracy of their shots, scoring more points for placements closer to the goalposts, areas that are less likely to be defended by goalkeepers, compared to shots directed towards the centre of the goal [17]. Similarly, Fortes et al. [15] found improvements in passing decision-making following an intervention in which participants watched offensive plays in VR that involved passing. In contrast, no significant differences between the VR intervention and the control groups were found on Stroop performance, a linguistic task that generally assesses cognitive inhibition. This finding raises some concern as to whether VR training, and cognitive training in general, can improve cognitive function in general or simply performance on the task that is trained [14] or tasks that are quite similar [15, 16, 17, 20]. It also poses an interesting question about context: do training tasks need to simulate the actual context of the sport?

On one hand, one could argue that the tasks athletes carry out in their sport rely on some core cognitive processes that could be amenable to training. For example, when reacting to a shot, a goalkeeper in football must engage in several processes relating to attention, e.g., maintain alertness, orient attention, and inhibit automatic responses. Indeed, studies have shown that individual differences in sports tasks can, in some cases, be accounted for by cognitive performance assessed through traditional tests. For example, Shimi et al. [8] found that performance on the Whack-a-Mole task, a computerized Go/No-Go task that requires participants to suppress a motor response, could predict goalkeeping performance in VR. Beyond behavioural inhibition assessed with the Whack-a-Mole task, Shimi et al. [8] provided evidence that phasic alertness, attentional orienting, and inhibitory control assessed with the Attentional Networks Test [22, 23] also explained a significant amount of variance in goalkeeping performance. Such results suggest that tasks that assess general cognitive function may capture mental skills that are relevant to real-life sports tasks and could potentially serve as effective training tools.

On the other hand, as shown in our short review, training tasks that simulate the environment of the sport, either through video [15] or graphics [16, 20], seem to have been the most effective. This suggests that perhaps context is important for training tools, as it allows athletes to improve performance by picking up the affordances of the environment and by strengthening stimulusresponse pairings. This could, down the line, lead to the automatic execution of actions when the athlete is in a real environment that matches well with that of training. Thus, contextualized VR training may offer an advantage by providing realistic practice environments that may improve transfer to real-world performance (see Craig [21] for a discussion on the topic).

A notable finding from our review is that benefits to performance seem to be present even when training does not entail the active execution of a task. Indeed, in the study by Fortes et al. [15], passing accuracy improved even when the players simply watched videos in VR without engaging into any passing activity themselves. This finding raises the question as to whether active engagement in a task during training is necessary to obtain benefits in performance. With regards to the specific study, one could argue that players did not improve the cognitive skills that underlie passing accuracy but instead they improved the technique by picking up information from the videos. Regardless of whether this was the case or not, a question for future research remains as to whether cognitive skills could be improved by simply watching others perform.

Another interesting possibility arising from our review is that the magnitude of training benefit may depend on athletes’ initial cognitive performance levels. Wilkerson et al. [19] demonstrated that players with lower baseline cognitive performance experienced greater improvements following VR interventions. In the study, the authors evaluated collegiate players, separating them into groups based on initial cognitive performance scores. After VR training, the lower-performing group showed a notable improvement from 68.6 to 87.7%, while the higher-performing group improved from 93.8 to 97.7%. Although this finding may be a consequence of a ceiling effect among high-performing athletes, it raises the question as to whether cognitive training is suitable for elite athletes with whom further improvements could be more difficult to achieve. Studies may further investigate the differential impacts of VR and AR training across performance levels, including elite athletes, and on tailoring the complexity of investigations to maximize their potential benefits.

Regarding the design of interventions, some studies have indicated that training of short duration with AR [14] or VR [20] can generate significant improvements. The study by Richlan [20] demonstrated that four weeks of VR training were sufficient to induce changes in brain function and structure. This finding suggests that VR may be effective even in the short term, with effects potentially lasting for some time (e.g., 4 weeks). These results open a new topic for investigating the minimum effective dose of VR training, including the optimal number and frequency of sessions. If short-term VR training proves effective, it could allow coaches to strategically implement specific VR sessions during specific phases of the sport season, such as pre-competition (e.g., *tapering*), when loads are reduced, to optimize athlete performance. It should be noted however that the findings of Bloechle et al. [14] and Fortes et al. [15] are at odds with those from similar studies carried out on basketball. Indeed, in a review of the literature on VR training in basketball, Chan et al. [24] concluded that the most effective VR-based tactical interventions used in basketball require long-term exposure. Given this discrepancy in the literature, future studies may compare VR interventions across sports. While soccer and basketball are both intermittent sports characterized by opponent interaction and rapid decision-making, VR interventions may need to differ in order to accommodate the specific sensorimotor and tactical demands of each sport and the way these are represented in virtual training environments.

### Limitations of the review

Although this review provides a potentially useful overview of the effects of VR and AR training on football players, some limitations must be acknowledged. First, the fact that only a small number of studies were identified raises the probability of the presence of publication bias. That is, given that studies with positive results (i.e., significant differences across intervention and control groups) are more easily published than those with null effects, it could be that the 7 studies we located on the topic represent false positives. However, given that studies in other sports have documented the presence of training benefits [25, 26], we consider this possibility rather unlikely. Still, some caution in drawing definitive conclusions seems warranted. Second, the studies we reviewed have rather small sample sizes. Although this is typical in studies with athletes, especially those at the elite level, the small sample sizes limit the statistical power and generalizability of the findings. What is more, the heterogeneity in both the types of interventions applied and in the dependent variables complicates direct comparisons and synthesis. Finally, most of the studies we reviewed did not include long-term follow-up assessments, making it difficult to evaluate the sustainability of the training effects over time. Addressing these limitations in future research will be essential to establish more definitive conclusions about the effectiveness of VR-based training tools.

## CONCLUSIONS

In conclusion, the findings of this systematic review support the potential of VR and AR training as an effective approach to improve cognitive and motor performance in soccer players. Despite the small number of studies on the topic, and while some caution regarding publication bias is warranted, the findings of the review suggest that VR and AR training can be effective across a range of measures. Overall, the outcomes of the review highlight the importance of designing context-specific training tasks, selecting appropriate evaluation measures, together with the cognitive and motor demands involved in players’ actions in real-world training and games. It is especially important to consider key domains of soccer performance where VR and AR may offer the greatest benefit, such as decisionmaking under pressure, visual scanning and set-piece execution.

Although short-term VR interventions show promising results, further studies are needed to establish standardized protocols, determine the duration of the training effect and explore applications in elite athlete populations. By addressing these areas, VR and AR technologies could become integral components of modern training strategies in soccer as well as different sports.

## References

[1] Mota T, Silva RM, Clemente F. Holistic soccer profile by position: a theoretical framework. Hum Mov. 2021; 24 (1):4–20.

[2] Pillitteri G, Giustino V, Petrucci M, Rossi A, Leale I, Bellafiore M, et al. Match load physical demands in U-19 professional soccer players assessed by a wearable inertial sensor. J Funct Morphol Kinesiol. 2013; 8(1):22. 10.3390/jfmk8010022 PMC9953515 36810506

[3] Scharfen HE, Memmert D. Measurement of cognitive functions in experts and elite athletes: A meta-analytic review. Appl Cogn Psychol. 2019; 33(5):843–860.

[4] Habekost T, Ovesen J, Madsen JB. Cognition in elite soccer players: a general model. Front Psychol. 2024; 15:1477262. 39723399 10.3389/fpsyg.2024.1477262 PMC11668572

[5] Aquino RL, Gonçalves LGC, Vieira LHP, Oliveira LP, Alves GF, Santiago PRP et al. Periodization training focused on technical-tactical ability in young soccer players positively affects biochemical markers and game performance. J Strength Cond Res. 2016; 30(10):2723–2732. 26890976 10.1519/JSC.0000000000001381

[6] Oliva-Lozano JM, Gómez-Carmona CD, Fortes V, Pino-Ortega J. Effect of training day, match, and length of the microcycle on workload periodization in professional soccer players: a full-season study. Biol Sport. 2022; 39(2):397–406. 35309541 10.5114/biolsport.2022.106148 PMC8919886

[7] Hannon MP, Coleman NM, Parker LJ, McKeown J, Unnithan VB, Close GL et al. Seasonal training and match load and micro-cycle periodization in male Premier League academy soccer players. J Sports Sci. 2021; 39(16):1838–1849. 33759688 10.1080/02640414.2021.1899610

[8] Shimi A, Tsestou V, Hadjiaros M, Neokleous K, Avraamides M. Attentional skills in soccer: Evaluating the involvement of attention in executing a goalkeeping task in virtual reality. Appl Sci. 2021; 11(19):9341.

[9] Shimi A, Papantoniou A, Neokleous K, Avraamides MN. Athletic performance in immersive virtual reality: the effect of training intensity. Eur J Psychol Open. 2022; 81(1):24–33.

[10] Thatcher B, Ivanov G, Szerovay M, Mills G. Virtual reality technology in football coaching: barriers and opportunities. Int Sport Coach J. 2020; 8(2):234–243.

[11] Garritty C, Gartlehner G, Nussbaumer-Streit B, King VJ, Hamel C, Kamel C, et al. Cochrane Rapid Reviews Methods Group Offers Evidence-Informed Guidance to Conduct Rapid Reviews. J Clin Epidemiol. 2021; 130:13–22. 33068715 10.1016/j.jclinepi.2020.10.007 PMC7557165

[12] Page MJ, McKenzie JE, Bossuyt PM, Boutron I, Hoffmann TC, Mulrow CD et al. The PRISMA 2020 Statement: An Updated Guideline for Reporting Systematic Reviews. BMJ. 2021; 372:71. 10.1136/bmj.n71 PMC8005924 33782057

[13] Ouzzani M, Hammady H, Fedorowicz Z, Elmagarmid A. Rayyan—a web and mobile app for systematic reviews. Syst Rev. 2016; 5:210. 27919275 10.1186/s13643-016-0384-4 PMC5139140

[14] Bloechle JL, Audiffren J, Le Naour T, Alli A, Simoni D, Wuthrich G, et al. It’s not all in your feet: Improving penalty kick performance with human-avatar interaction and machine learning. Innovation.2024; 5(2):100584. 38445019 10.1016/j.xinn.2024.100584 PMC10912701

[15] Fortes LS, Almeida SS, Praça GM, Nascimento-Júnior JR, Lima-Junior D, Barbosa B, et al. Virtual reality promotes greater improvements than video-stimulation screen on perceptual-cognitive skills in young soccer athletes. Hum Mov Sci. 2021; 79:102856. 34391110 10.1016/j.humov.2021.102856

[16] Gürbüz E, Taş M. The effect of virtual reality training on heading skills in 12–13 years old child footballers. Spor Bilimleri Araştırmaları Dergisi, 2023; 8(1):43–56.

[17] Marshall B, Uiga L, Parr JVV, Wood G. A preliminary investigation into the efficacy of training soccer heading in immersive virtual reality. Virtual Real. 2023; 27(3):2397–2404.

[18] Rusmanto R, Tomoliyus T, Sulastion A, Gazali N, Abdullah KH, Espinosa FJG et al. Virtual reality to promoting sports engagement and some technical skills in junior football athletes: A 12-week randomized controlled trial. Retos.2023; (50):1129–1133.

[19] Wilkerson GB, Mether KS, Perrin ZA, Emberton SL, Carlson LM, Hogg JA et al. Perceptual response training for reduction of injury risk among high school girls’ soccer players. Brain Sci. 2024; 14(11):1091. 39595854 10.3390/brainsci14111091 PMC11592295

[20] Richlan F. Behavioral and neuroanatomical effects of soccer heading training in virtual reality: a longitudinal fMRI case study. Neuropsychologia. 2025; 211:109124. 40089102 10.1016/j.neuropsychologia.2025.109124

[21] Craig C. Understanding perception and action in sport: how can virtual reality technology help? Sports Technol. 2014; 6 (4):161–169.

[22] Fan J, McCandliss BD, Sommer T, Raz A, Posner MI. Testing the efficiency and independence of attentional networks. J. Cogn. Neurosci. 2002; 1:340–347. 10.1162/089892902317361886 11970796

[23] Rueda MR, Fan J, McCandliss BD, Halparin JD, Gruber DB, Lercari LP et al. Development of attentional networks in childhood. Neuropsychologia 2004; 42:1029–1040. 15093142 10.1016/j.neuropsychologia.2003.12.012

[24] Chan CHM, Ma MKI, Chan TS, Yeung LT, Chan K. Current applications of virtual reality in basketball training: a systematic review. Sports Engineering. 2024; 27:26.

[25] Witte K, Droste M, Ritter Y, Emmermacher P, Masik S, Bürger D et al. Sports training in virtual reality to improve response behavior in karate kumite with transfer to real world. Front Virtual Real. 2022; 3:903021.

[26] Gray R. Transfer of training from virtual to real baseball batting. Front Psychol. 2017; 8:317328. 10.3389/fpsyg.2017.02183 PMC5733365 29326627

